# Effect of Er:YAG Laser Irradiation and Acidulated Phosphate Fluoride Therapy on Re-Mineralization of White Spot Lesions

**DOI:** 10.30476/DENTJODS.2020.86300.1187

**Published:** 2021-09

**Authors:** Hossein Assarzadeh, Malihe Karrabi, Reza Fekrazad, Yaser Tabarraei

**Affiliations:** 1 Dept. of Oral and Prosthodontics, School of Medicine, Sabzevar University of Medical Sciences, Sabzevar, Iran; 2 Dept. of Periodontology, Dental Faculty, Laser Research Center In Medical Sciences, AJA University of Medical Sciences, Tehran, Iran; 3 Radiation Science Research Center, Laser Research Center in Medical Science, AJA University of Medical Sciences, Tehran, Iran; 4 International Network for Photo Medicine and Photo Dynamic Therapy (INPMPDT), University Scientific Education and Research Network (USERN), Tehran, Iran; 5 Dept. of Epidemiology and Biostatistics, School of Public Health, Tehran University of Medical Sciences, Tehran, Iran

**Keywords:** Er:YAG laser, Fluoride ion, Remineralization, White spot lesion

## Abstract

**Statement of the Problem:**

Studies on the efficacy of erbium laser for enhancement of enamel resistance to acid attacks and its effects on fluoride uptake by the enamel are limited.

**Purpose:**

This study sought to assess and compare the effects of erbium-doped yttrium aluminum garnet (Er:YAG) laser irradiation and application of acidulated phosphate fluoride (APF) gel (alone and in combination) on remineralization of artificial white spot lesions (WSLs).

**Materials and Method:**

This *in vitro*, experimental study evaluated 90 buccal and lingual slabs of extracted human premolars. The specimens underwent pH cycling to induce WSLs. They were then randomly divided into 6 groups of caries-free positive control (c+), negative control with WSLs (ws), 1.23% APF gel applied on the enamel (F), Er:YAG laser irradiation (80 mJ, 10 Hz, and 8 J/cm^2^) of enamel (L), APF gel application followed by laser irradiation (FL), and laser irradiation followed by fluoride gel application (LF). The fluoride ion content of specimens was measured before and after the intervention using a potentiometer. Data were analyzed by ANOVA (*p*< 0.05).

**Results:**

APF gel application before/after laser irradiation maximally increased the fluoride uptake by the enamel (*p*= 0.000). Application of APF gel in group F and laser irradiation in group L increased fluoride uptake by the enamel, compared with groups 1 and 2 (*p*= 0.000). Laser- treated (L) and APF-treated (F) groups had no significant difference in this respect (*p*= 0.945). Maximum fluoride concentration was noted in combined laser and fluoride groups (FL=332.07ppm and LF=341.27ppm) with no significant difference between the two (*p*= 1.000).

**Conclusion:**

Er:YAG laser irradiation changes the chemical composition of enamel and probably promote its remineralization, especially when combined with APF gel application, which highlights its cariostatic potential.

## Introduction

Nowadays, modern caries management focuses on non-cavited carious lesions, preventing caries progression and their remineralization instead of drilling of tooth structure and filling. Recently, there have been many innovative advances in enamel remineralization promotion. Some of these methods are independent of fluoride therapy, such as tooth regeneration via dentin phosphoprotein. This material can promote remineralization of the tooth surface when it is present in a solution including calcium and phosphate, just like saliva [ 1 - 2 ]. Moreover, modern systems such as recombinant porcine amelogenin (rP172) and leucine-rich amelogenin peptide, containing amelogenin (the protein that regulates the growth and maturation of enamel crystals in newly formed enamel matrix) stabilize calcium phosphate to promote crystal formation and direct mineral growth [ 3 - 4 ]. Nano hydroxyapatite is an important bioactive material that has ability to enhance enamel remineralization. Hence, these nano-sized particles can repair enamel surface via binding strongly to the enamel surface and filling up the holes and gaps of it [ 5 ]. However, it has been well documented that fluoride therapy could be considered as the cornerstone and best method for non-invasive caries management and remineralizing of white spot lesions (WSLs) [ 6 - 7 ].

Different materials can be used for fluoride delivering into WSL such as sodium fluoride (NAF-1,450 ppm), acidulated phosphate fluoride (APF-12,300 ppm) and MI paste plus (containing casein phosphopeptideamorphous calcium phosphate with fluoride compound-900ppm). Higher concentration of the fluoride leads to more fluoride uptake; improve enamel crystal development and more transformation of hydroxyapatite crystal into fluorapatite. APF gel is the most commonly used product for professional fluoride therapy. Calcium fluoride (CaF_2_) is the main product of the reaction of APF with the enamel that deposits on the enamel surface and the subsurface area of the enamel carious lesions [ 8 ]. Although topical application of fluoride is effective for prevention of WSLs, it is not an efficient method for treatment and control of these lesions, because deposited fluoride may be lost again *in vivo* by back diffusion, back exchange, and migration from the mineral to the surrounding tissue fluid, saliva, or plaque fluid [ 9 ]. Therefore, reservoir-releasing fluoride decreases after short periods. Some strategy has been suggested to overcome this problem, such as several applications of topical fluoride, or increasing fluoride uptake by laser irradiation [ 10 ].

Recently, lasers are used for caries prevention due to their significant effects on dental hard tissue [ 11 ]. Laser irradiation increases the enamel acid resistance by mechanisms such as fusion, changing the crystallinity, and decreasing the permeability of enamel to chemical agents [ 12 - 13 ]. Laser irradiation increases the size of hydroxyapatite crystals by melting, causes recrystallization of enamel, and subsequently decreases the permeability of enamel and enhances its resistance to acid attacks [ 14 ]. Evidence shows that erbium laser is highly capable of enamel removal, and its radiation can prevent caries development by decreasing the microorganism count and causing chemical and morphological changes in the enamel structure [ 15 - 16 ]. 

Studies on the efficacy of erbium laser for enhancement of enamel resistance to acid attacks and its effects on fluoride uptake by the enamel are limited. Thus, this study sought to assess and compare the effects of erbium-doped yttrium aluminum garnet (Er: YAG) laser and APF gel on remineralization of WSLs.

## Materials and Method

This *in vitro*, experimental study assessed the fluoride uptake of 90 specimens (n=15 in each group) after different surface treatments by potentiometry and use of a fluoride selective electrode. The Ethics Committee of Shahid Sadoughi University of Medical Sciences (IR.SSU.REC.1399.18013) approved this study.

A total of 45 sound human premolars extracted for orthodontic treatment were used in this study. The teeth were immersed in 0.1% thymol solution at a pH of 7 at room temperature (25°C) until the experiment [ 17 ]. For preparation of specimens, the buccal and lingual enamel surfaces were ground using 400 and 800-grit abrasive finishing discs (Finishing Disc Kit, Bisco, USA). This was done to eliminate the superficial enamel layer with possibly high fluoride content and for standardization of samples. The roots were then cut and the lingual and buccal surfaces of each tooth were cut mesio-distally with 2 mm thickness. Thus, 90 buccal and lingual slabs were obtained as such. After 30 seconds of rinsing with distilled water and drying the specimens, all slabs were inspected under a stereomicroscope. The specimens with cracks and structural defects were excluded. Fifteen specimens were randomly selected as the positive control group (C+). Next, the entire surface of 75 specimens, except for a circle with 5 mm diameter at the center of ground enamel surface, was coated with acid-resistant nail varnish (Kenvis, UAE). Afterwards, the specimens were immersed in a demineralizing solution composed of 2.2 mM CaCl_2_, 2.2 mM NaH_2_PO_4_, and 50 mM acetic acid (pH of 4.8) for 10 weeks to induce WSLs. This solution was changed weekly [ 18 ]. After completion of demineralization, all specimens were visually inspected by the same operator for detection of WSLs. The specimens with surfaces that appeared normal in presence of water but showed an opaque chalky appearance in absence of water were separated and examined by DIAGNOdent (Kavo, Germany) to ensure the development of WSLs. 

The device was calibrated against a proprietary ceramic standard before each measurement. Scores 14-20 displayed by the device confirmed the presence of WSLs [ 19 - 20 ]. Next, the specimens were randomly divided into 5 groups of 15each. The negative control group (WS) was previously separated. The study groups have been shown at Table 1.

**Table 1:** Study Groups

Group	Intervention
1) C+	Positive control group
2) WS	Negative control group with WSLs
3) F	WSLs subjected to APF gel application
4) L	WSLs subjected to Er:YAG laser irradiation
5) FL	WSLs subjected to APF gel application followed by Er:YAG laser irradiation
6) LF	WSLs subjected to Er:YAG laser irradiation followed by fluoride gel application

### Fluoride gel application

For F (APF gel) group, APF gel (Sultan Chemist, Englewood NJ, USA) containing 1.23% fluoride was applied on the surface by a cotton roll in 1-2mm thickness for 4 min and was then washed thoroughly using deionized water for one minute to remove any visible remnants of the gel and dried [ 20 - 21 ]. 

### Laser irradiation

For L (laser) group, Er:YAG laser (Key III, Kavo, Germany), with the exposure settings of 80 mJ, 10 Hz, and 8J/cm^2^, with a wavelength of 2940 nm and spot size of 0.63mm was irradiated in non-contact mode without air and water spray. Laser irradiation was done in pulsed emission mode, by fiber Optic delivery system with the sapphire tip (Ø1.0mm), pulse duration of 250–500 mSec, applied with a no. 2051 hand piece. Laser was irradiated manually for 10 s by scanning movement at 5 mm distance using a hand-made jig to scan the entire exposed enamel surface [ 22 ]. Before of use, The energy levels were calibrated by calibrating the tip of the Erbium fiber with the special proprietary tip calibration handpiece, according to the manufacturer’s instructions. The energy delivered was measured periodically with a P, Coherent; Santa Clara, CA, USA). For LF (laser irradiation followed by fluoride gel application( and FL group (APF gel application followed by laser irradiation), laser irradiation was done similar to L group, and fluoride application was done similar to F group. Then the specimens were stored for 24h in sealed polyethylene test tube containing deionized water, before the tests were conducted.

### Measuring the fluoride concentration

The fluoride ion concentration was measured by the enamel biopsy technique using a potentiometer (Metrohm, Switzerland) and a fluoride selective electrode (Fluoride ISE Metrohm 2000, Switzerland). Each specimen was independently immersed in a beaker containing 10 cc of 0.5 molar per chloric acid (HClO_4_) and removed after 30 s by a hemostat. Next, the enamel surface was rinsed with 20 cc of 0.2 molar KOH. By doing so, 30 mL of biopsy solution was obtained. For measurement of fluoride concentration, EDTA was added to the solution to adjust the pH at 5.2 using a pH meter (780 pH Meter, Metrohm, Switzerland).

Certain concentrations of sodium fluoride salt were prepared and their potential difference was measured by a potentiometer. The potential difference-concentration graph was drawn by a computer program and the respective linear equation was extracted accordingly. The beaker was then placed on the magnet and the standard electrodes of potentiometer along with the fluoride selective electrode were placed in the solution, and the potential difference of each solution was recorded. The potential difference value was used in the linear equation derived from the potential difference-concentration graph, and the fluoride concentration of each specimen was quantified as such [ 23 ].

A blinded operator performed all of the study steps include specimens preparation, fluoride application, laser irradiation, the laser fluorescence readings and measuring the fluoride concentration according to the manufacturaer’s instructions.

Data were analyzed using ANOVA at 95% confidence interval.

## Results

Table 2 shows the minimum, maximum, mean and standard deviation of fluoride concentration in the study groups. Maximum fluoride concentration was noted in group LF (Laser-APF group with mean concentration of 341.27 ppm) while minimum fluoride concentration was noted in group WS (White spot group with mean concentration of 16.20ppm). Among intervention groups, minimum concentration of fluoride was observed in L group. The ANOVA test revealed a significant difference in the mean concentration of fluoride between the groups at 95% confidence interval (*p*= 0.00). The Tukey’s post-hoc test was used for pairwise comparisons of the groups, and the results are presented in Table 3. As shown in Table 3 and Table 4, the difference between groups C+ and WS (*p*= 0.997), groups F and L (*p*= 0.945), and groups FL and LF (*p*= 1.000) was not significant. However, the difference between groups C+ and WS with groups F and L and also FL and LF was significant. Groups F and L also had significant differences with groups FL and LF (*p*= 0.000).

**Table 2:** Mean and standard deviation of fluoride concentration in the study groups

Group	N	MIN	MAX	Means ±SD
1) Control	15	13	81	31.46±1.66
2) White spot	15	11	36	16.20±7.41
3) APF	15	111	328	231.67±6.40
4) Laser	15	99	365	201.53±7.49
5) APF- laser	15	111	573	332.07±129.17
6) Laser-APF	15	191	603	341.27±153.86

**Table 3:** Tukey post hoc test comparing each group with all other ones

Group	Mean Difference	*p*	95% CI
Control versus white spot	15.26	.997	-82.36-112.89
Control versus laser	-170.06*	.000	-267.69- -72.43
Control versus APF	-200.20*	.000	-297.82--102.57
Control versus APF- laser	-300.60*	.000	-398.22--202.97
Control versus laser- APF	-309.80*	.000	-407.42--212.17
white spot versus laser	-185.33*	.000	-282.96- - 87.70
white spot versus APF	-215.46*	.000	-313.09--117.83
white spot versus APF-laser	-315.86*	.000	-413.49--218.23
white spot versus laser-APF	-325.06*	.000	-422.69--227.43
Laser versus APF	-30.13	.945	-127.76- 67.49
Laser versus APF- laser	-130.53*	.003	-228.16--32.90
Laser versus laser- APF	-139.73	.001	-237.36-- 42.10
APF versus APF- laser	-100.40*	.040	-198.02--2.77
APF versus laser- APF	-109.60	.019	-207.22-- 11.97
APF- laser versus laser	130.53*	.003	32.90- 228.16
APF-laser versus laser-APF	-9.20	1.000	-106.82-88.42

**Table 4:** Means for groups in homogeneous subsets

Group	N	Subset for alpha= 0.05		
		1	2	3
C+	15	16.20		
WS	15	31.46		
F	15		231.67	
L	15		201.53	
FL	15			332.07
LF	15			341.27
Sig.		.997	.945	1.000

## Discussion

According to the yielded results, the fluoride concentration of enamel has been increased following application of APF gel and Er: YAG laser irradiation. It means that laser irradiation was as effective as fluoride gel application in increasing the fluoride concentration of the enamel. Combined use of both modalities caused a significant increase in fluoride concentration of the enamel. Moreover, according to the results, the order of using laser and fluoride had no significant effect on the results.

WSLs develop as the result of long-term accumulation of bacterial plaque and subsequent acidic dissolution of the enamel. Several models are available for induction of WSLs. In the present study, pH cycling was performed to simulate the oral environment. This model involves the use of remineralizing and demineralizing solutions, and simultaneously measures the outcome of inhibition of demineralization and enhancement of remineralization. It simulates the changes in the oral environment after food intake [ 24 ]. In addition to visual examination, which is the main technique of examination in the clinical setting for detection of WSLs, some adjuncts are also available for this purpose such as the use of DIAGNOdent, which operates based on the laser fluorescence technology [ 20 ]. In this study, visual examination was used along with DIAGNOdent to ensure the development of WSLs after pH cycling and in order to convert a qualitative variable to a quantitative variable and for resultantly higher validity and reliability. The specimens that showed score 14-20 were considered as having WSLs according to the manufacturer’s instructions [ 20 ].

Furthermore, in this study, the teeth in each group were split into buccal and lingual halves. It has been shown that symmetrical areas in one tooth have the same fluoride content, and this method decreases the possibility of errors due to the different baseline fluoride contents of different teeth [ 19 ].

Minimum concentration of fluoride was noted in group WS, that had WSLs following pH cycling and received no intervention, which is probably due to mineral loss of the enamel as the result of immersion in demineralizing solution. However, in group F, application of APF gel increased the fluoride concentration of the enamel. Fluoride can prevent demineralization of sound enamel by interfering with the microbial flora producing glucose transferase enzyme [ 25 - 26 ]. Fluoride also promotes enamel remineralization by participation in the enamel structure and increasing the deposition of Fluor apatite on the tooth structure [ 27 ]. The former mechanism is the main remineralization mechanism in this study. 

Decreased solubility and increased fluoride uptake by the enamel in L (laser group) can be due to the sub-ablative effects of Er:YAG laser, change in chemical composition of enamel, and formation of Fluor apatite crystals [ 28 - 29 ]. Several theories have been suggested regarding the mechanism of increasing the enamel resistance to acid attacks by laser irradiation. These include decreasing the enamel permeability by melting and recrystallization of enamel surface, decreasing the enamel solubility by formation of particles with lower solubility such as monoxide tetra calcium diphosphate, and decreased water and carbonate content and increased hydroxyl ion content, formation of pyrophosphate, and decomposition of proteins [ 30 ]. In this study, Er:YAG laser irradiation with 80 mJ energy intensity in group L increased the concentration of fluoride ions compared with the positive and negative control groups (C+ and WS). 

Evidence shows that use of water and air coolant during laser irradiation can negatively affect its caries-prevention efficacy because the laser energy is absorbed by water and becomes less effective [ 15 , 31 ]. Thus, laser was irradiated without air and water coolant in this study to achieve the desired temperature (>100°C) because the carbonate loss starts after raising the temperature to 100°C. This temperature raising has just occurred in enamel surface so the possibility of pulpal damage has not been significant.

Evidence shows an association between carbonate loss and increased enamel resistance to acid attacks [ 23 - 32 ]. Since carbonate has less adaptation to enamel crystals, the crystals are reoriented in a more stable and more resistant form after carbonate loss [ 33 - 34 ]. Moreover, the critical enamel pH is 5.5, which decreases to 4.8 following laser irradiation [ 12 ]. This finding can be interpreted by noticing the fact that irradiated laser is absorbed by some certain components, and the radiation energy is directly converted to heat. This would cause structural and chemical crystallographic changes in the enamel and increases its resistance to acid attacks [ 34 ]. The oxygen and phosphate contents of the enamel significantly increase after laser irradiation due to the increased pyrophosphate content following enamel heating [ 13 ]. After laser irradiation, the calcium (Ca) content increases, this subsequently increases the Ca/P ratio especially when higher energy densities are used. Under such circumstances, calcium is released in lower amounts following acid exposure [ 13 ].

The effects of different laser temperatures on the enamel structure have been previously studied [ 35 - 36 ]. Laser at 100°C to 650°C and low energy (0.3 J) causes oxidation of organic materials, converts acidulated phosphate to pyrophosphate, and results in loss of water and carbonate from the enamel structure [ 35 - 37 ]. Due to the generated heat and melting phenomenon, some compounds such as tetra calcium diphosphate monoxide, alpha tricalcium phosphate (α-TCP) and beta-tricalcium phosphate (β-TCP) are formed. This change in the organic composition of the enamel can lead to obstruction of prisms and decreased permeability [ 38 ]. At 650°C-1100°C, decomposition and oxidation of carbonate occur. At 1100°C, all the carbonate content is lost, and a new crystalline phase of alpha tricalcium phosphate and beta-tricalcium phosphate forms, which is resistant to demineralization [ 39 - 40 ]. 

Laser application along with fluoride treatment is a novel technique to improve fluoride uptake to the enamel structure. Different laser types have been used to serve this purpose, such as CO_2_, Diode Laser, Er:YAG and Nd:YAG laser [ 22 , 28 ]. It has been shown that the application of CO_2_ laser or diode laser in combination with topical fluoridation inhibits the damage caused by acid attack [ 22 ]. CO_2_ laser irradiation combined with fluoride reduces the enamel solubility in acidic conditions and increases fluoride uptake [ 23 ]. 

Despite the fact that Er:YAG lasers are mainly used for hard tissue ablation, limited studies have reported that use of low-energy Er:YAG laser can lead to caries prevention [ 15 ]. Liu and Hsu [ 41 ] stated that reduction in carbonate content and change in organic composition of the enamel are the main caries prevention mechanisms of Er:YAG laser, which is achieved by prevention of enamel demineralization without dental tissue ablation or morphological damage to the enamel surface. The ablation range of Er:YAG laser is between 9 to 11 J/cm^2^ [ 42 ]. High laser energy in each pulse and higher frequency of pulses per second would result in greater enamel resistance to demineralization [ 43 ]. 

In this study, Er:YAG laser was used with 80 mJ energy, 10 Hz repetition rate, and 8J/cm^2^ energy density in non-contact mode. Laser was irradiated from 5 mm distance using a hand-made jig in order to prevent enamel ablation. The current results were in agreement with the findings of other studies and showed an increase in fluoride concentration and micro-hardness of initially demineralized enamel surfaces following laser irradiation [ 13 , 30 , 44 ]. Moreover, it has been claimed that cavity preparation by this laser increases the resistance to demineralization and prevents secondary caries [ 45 - 46 ]. 

In this study, maximum fluoride concentration was noted in groups FL and LF, where a combination of laser irradiation and fluoride therapy was used. Increased fluoride concentration in these groups could be due to the increased accumulation and stability of calcium fluoride formed on the enamel surface. Scanning electron microscopic studies have shown increased deposition of spherical or globular deposits measuring 2-4 µ in size with morphology similar to that of calcium fluoride. Calcium fluoride can serve as a fluoride reservoir against acid attacks [ 13 ]. The primary calcium fluoride deposit on the enamel surface subjected to fluoride application alone is not stable, and its fluoride content is quickly lost within the first 24 hours and this process continues for 15 days. In fact, laser increases the fluoride uptake in the form of firmly bound fluoride [ 47 ]. (4, 6) It seems that physical, chemical and kinetic changes that occur following laser irradiation can increase the penetration of fluoride and its stabilization in the enamel structure. Due to the generation of heat energy and melting points in some parts of the enamel surface, fluoride penetrates into the melted crystals and forms new compounds after cooling of enamel. Stability of fluoride in the enamel surface in long-term due to the changed polarity of crystals and increased uptake and influx of fluoride into the crystals after the loss of water and carbonate can explain the boosting effect of laser irradiation on the already optimal efficacy of fluoride therapy [ 14 ]. Simultaneous use of laser and fluoride enhances the penetration of fluoride by up to 14 times and to 20µ depth into the crystals [ 48 ]. 

Bevilacqua *et al*. [ 30 ] assessed the effect of erbium laser using spectrophotometry and atomic absorption spectrometry and concluded that Er:YAG laser with 1.8 J/cm^2^ and 0.9 J/cm^2^ fluencies decreased acid solubility and increased fluoride uptake. Combined use of laser and topical fluoride had a greater efficacy for prevention of enamel demineralization and lower decrease in surface micro hardness [ 20 , 23 , 30 ]. In this study, the mean concentration of fluoride in groups FL and LF was higher than in other groups, which indicates that combined therapy was more effective for enhancement of enamel resistance to acid attacks.

Use of fluoride compounds along with laser irradiation yields an enamel structure, which is more resistant to caries and decreases the unfavorable changes caused by laser irradiation, since it prevents irreparable and unfavorable alterations in the enamel structure [ 49 - 50 ].

It should be noted that due to the high absorption of Er:YAG laser by the water in the composition of enamel, laser irradiation causes micro-explosions and subsequent ablation. This leads to formation of an irregular enamel surface that enhances plaque accumulation. Thus, this laser should be used in sub-ablative conditions to improve chemical alterations and decrease morphological changes of the enamel surface [ 30 , 46 ].

Further, *in vivo* and *in vitro* studies with a larger sample size using profilometry and chemical assessment are necessary to investigate the effect of laser in reduction of the progression of caries lesions in long time.

## Conclusion

Irradiation of sublative Er:YAG laser before/after APF fluoride therapy probably changes the chemical structure of the enamel, and increases fluoride uptake and decreases its permeability. It seems that Er:YAG laser could be helpful for prevention of caries development and inhibition of WSL progress. Thus, it can be used along with fluoride therapy as an effective and safe modality for remineralization of WSLs in the clinical setting. Combined use of fluoride and Er:YAG laser irradiation has a synergistic effect compared with the use of each modality alone, and further enhances the fluoride uptake. Moreover, irradiation of Er:YAG laser before/after APF fluoride therapy does not have any effect on amount of fluoride uptake.

## References

[1] Farooq I, Bugshan A ( 2020). The role of salivary contents and modern technologies in the remineralization of dental enamel: a narrative review. F1000Res.

[2] Prasad M, Butler WT, Qin C ( 2010). Dentin sialophosphoprotein in biomineralization. Connective Tissue Res.

[3] Fan Y, Sun Z, Moradian-Oldak J ( 2009). Controlled remineralization of enamel in the presence of amelogenin and fluoride. Biomaterials.

[4] Le Norcy E, Kwak SY, Wiedemann-Bidlack FB, Beniash E, Yamakoshi Y, Simmer JP, et al ( 2011). Leucine-rich amelogenin peptides regulate mineralization in vitro. J Dent Res.

[5] Philip N ( 2019). State of the Art Enamel Remineralization Systems: The Next Frontier in Caries Management. Caries Res.

[6] Wang H, Xiao Z, Yang J, Lu D, Kishen A, Li Y, et al ( 2017). Oriented and Ordered Biomimetic Remineralization of the Surface of Demineralized Dental Enamel Using HAP@ACP Nanoparticles Guided by Glycine. Sci Rep.

[7] Premnath P, John J, Manchery N, Subbiah GK, Nagappan N, Subramani P ( 2019). Effectiveness of theobromine on enamel remineralization: a comparative in vitro study. Cureus.

[8] Duraisamy V, Xavier A, Nayak UA, Reddy V, Rao AP ( 2015). An in vitro evaluation of the demineralization inhibitory effect of F(-) varnish and casein phosphopeptide-amorphous calcium phosphate on enamel in young permanent teeth. J Pharm Bioallied.

[9] Dijkman AG, Boer P, Arends J ( 1983). In vivo Investigation on the Fluoride Content in and on Human Enamel after Topical Applications. Caries res.

[10] Jahanimoghadam F, Poureslami H, Shamsaddin H, Horri A, Khazaeli P, Mahvi A ( 2016). Effect of Er:YAG laser on sodium fluoride varnish uptake by primary tooth enamel: An in vitro study. Fluoride.

[11] Rezaei Y, Bagheri H, Esmaeilzadeh M ( 2011). Effects of Laser Irradiation on Caries Prevention. J Lasers Med Sci.

[12] Tepper SA, Zehnder M, Pajarola GF, Schmidlin PR ( 2004). Increased fluoride uptake and acid resistance by CO_2_ laser-irradiation through topically applied fluoride on human enamel in vitro. J Dent.

[13] Chin-Ying SH, Xiaoli G, Jisheng P, Wefel JS ( 2004). Effects of CO_2_ laser on fluoride uptake in enamel. J Dent.

[14] Paryab M ( 1389). Laser and enamel resistance to caries. Lasers in Medicine.

[15] Hossain M, Nakamura Y, Yuichi Kimura, Yamada Y, Ito M, Matsumoto K ( 2000). Caries-preventive effect of Er:YAG laser irradiation with or without water mist. J Clin Laser Med Surg.

[16] Lombardo G, Pagano S, Cianetti S, Capobianco B, Orso M, Negri P, et al ( 2019). Sub-ablative laser irradiation to prevent acid demineralisation of dental enamel. A systematic review of literature reporting in vitro studies. EJPD.

[17] Fornaini C, Riceputi D, Lupi-Pegurier L, Rocca JP ( 2012). Patient responses to Er:YAG laser when used for conservative dentistry. Lasers Med Sci.

[18] Heravi F, Ahrari F, Mahdavi M, Basafa S ( 2014). Comparative evaluation of the effect of Er:YAG laser and low level laser irradiation combined with CPP-ACPF cream on treatment of enamel caries. J Clin Exp Dent.

[19] Tveit AB ( 1980). Fluoride uptake by enamel surfaces, root surfaces and cavity walls following application of a fluoride varnish in vitro. Caries Res.

[20] González-Soteloa A, Rodríguez-Vilchisb LE, Contreras-Bulnesb R ( 2019). Effect of Er:YAG and Fluoride Varnishes for Preventing Primary Enamel Demineralisation. Oral Health Prev Dent.

[21] Molaasadollah F, Asnaashari M, Abbas FM, Fafary M ( 2017). In vitro comparison of flouride gel alone and in combination with Er,Cr:YSGG Laser on reducing white spot lesion in primary teeth. J Lasers Med Sci.

[22] Yassaei S, Motallaei MN ( 2020). The Effect of the Er:YAG Laser and MI Paste Plus on the Treatment of White Spot Lesions. J Lasers Med Sci.

[23] Bahrololoomi Z, Ardakani FF, Sorouri M ( 2015). In vitro comparison of the effects of diode laser and CO_2_ Laser on topical fluoride uptake in primary teeth. JD (Tehran).

[24] Haghgoo R, Haghgou HR, Abbasi F, Tavakkoli M ( 2015). The effect of nano-hydroxyappatite solution on the permanent tooth remineralization following exposure to soft beer (in situ). J Dental Med.

[25] Ccahuana-Vasquez RA, Tabchoury CP, Tenuta LM, Del Bel Cury AA, Vale GC, Cury JA ( 2007). Effect of frequency of sucrose exposure on dental biofilm composition and enamel demineralization in the presence of fluoride. Caries Res.

[26] Summitt JB, Robbins JW, Hilton TJ, Schwartz RS, Dos Santos Jr J (2006). Fundamentals of operative dentistry: a contemporary approach.

[27] Cochrane NJ, Cai F, Huq L, Burrow M, Reynolds E ( 2010). New Approaches to Enhanced Remineralization of Tooth Enamel. J Dent Res.

[28] Apel C, Meister J, Schmitt N, Gräber HG, Gutknecht N ( 2002). Calcium solubility of dental enamel following sub-ablative Er:YAG and Er:YSGG laser irradiation in vitro. LIMS.

[29] Zamudio-Ortega CM, Contreras-Bulnes R, Scougall-Vilchis RJ, Morales-Luckie RA, Olea-Mejía OF, Rodríguez-Vilchis LE, et al ( 2014). Morphological and Chemical Changes of Deciduous Enamel Produced by Er:YAG Laser, Fluoride, and Combined Treatment. Photomed Laser Surg.

[30] Bevilacqua FM, Zezell DM, Magnani R, da Ana PA, Eduardo Cde P ( 2008). Fluoride uptake and acid resistance of enamel irradiated with Er:YAG laser. Lasers Med Sci.

[31] Molla Asadollah F, Mojahedi SM, Nojedehian H, Asnaashari M, Asnaashari N ( 2019). The Effect of Er:YAG Laser Irradiation Combined With Fluoride Application on the Resistance of Primary and Permanent Dental Enamel to Erosion. J Lasers Med Sci.

[32] Nair AS, Kumar RK, Philip ST, Ahameed SS, Punnathara S, Peter J ( 2016). A Comparative Analysis of Caries Inhibitory Effect of Remineralizing Agents on Human Enamel Treated With Er:YAG Laser: An In-vitro Atomic Emission Spectrometry Analysis. J Clin Diagn Res.

[33] Oho T, Morioka T ( 1990). A Possible Mechanism of Acquired Acid Resistance of Human Dental Enamel by Laser Irradiation. Caries res.

[34] Apel C, Meister J, Gotz H, Duschner H, Gutknecht N ( 2005). Structural changes in human dental enamel after subablative erbium laser irradiation and its potential use for caries prevention. Caries Res.

[35] Fowler BO, Kuroda S ( 1986). Changes in heated and in laser-irradiated human tooth enamel and their probable effects on solubility. Calcif Tissue Int.

[36] Bevilácqua FM, Zezell DM, Magnani R, da Ana PA, Eduardo Cde P ( 2008). Fluoride uptake and acid resistance of enamel irradiated with Er:YAG laser. J Lasers Med Sci.

[37] Featherstone J, Fried D, Bitten E Mechanism of laser-induced solubility reduction of dental enamel. https://www.semanticscholar.org/paper/Mechanism-of-laser-induced-solubility-reduction-of-Featherstone-Fried/39616c3abac01b5e925550c5d60275bbd5c92e01.

[38] Bachmann L, Craievich A, Zezell D ( 2004). Crystalline structure of dental enamel after Ho:YLF laser irradiation. Arch Oral Biol.

[39] Rodrigues LK, Nobre dos Santos M, Pereira D, Assaf AV, Pardi V ( 2004). Carbon dioxide laser in dental caries prevention. J Dent.

[40] Fowler BO, Kuroda S ( 1986). Changes in heated and in laser-irradiated human tooth enamel and their probable effects on solubility. Calcif Tissue Int.

[41] Liu Y, Hsu CY ( 2007). Laser-induced compositional changes on enamel: a FT-Raman study. J Dent.

[42] Apel C, Meister J, Ioana RS, Franzen R, Hering P, Gutknecht N ( 2002). The ablation threshold of Er:YAG and Er:YSGG laser radiation in dental enamel. Lasers Med Sci.

[43] Yassaei S, Shahraki N, Aghili H, Davari A ( 2014). Combined effects of Er: YAG laser and casein phosphopeptide-amorphous calcium phosphate on the inhibition of enamel demineralization: An in vitro study. Dent Res J (Isfahan).

[44] Fekrazad R, Najafi A, Mahfar R, Namdari M, Azarsina M ( 2017). Comparison of enamel remineralization potential after application of titanium tetra fluoride and carbon dioxide laser. Laser Ther.

[45] Abbasi M, Nakhostin A, Namdar F, Chiniforush N, Hasani Tabatabaei M ( 2018). The Rate of Demineralization in the Teeth Prepared by Bur and Er:YAG Laser. J Lasers Med Sci.

[46] Colucci V, de Souza Gabriel AE, Scatolin RS, Serra MC, Corona SA ( 2015). Effect of Er:YAG laser on enamel demineralization around restorations. Lasers Med Sci.

[47] Tepper SA, Zehnder M, Pajarola GF, Schmidlin PR ( 2004). Increased fluoride uptake and acid resistance by CO_2_ laser-irradiation through topically applied fluoride on human enamel in vitro. J Den.

[48] Zhang C, Kimura Y, Matsumoto K ( 1996). The effects of pulsed Nd:YAG laser irradiation with fluoride on root surface. J Clin Laser Med Surg.

[49] Nammour S, Demortier G, Florio P, Delhaye Y, Pireaux JJ, Morciaux Y, et al ( 2003). Increase of enamel fluoride retention by low fluence argon laser in vivo. Laser Surg Med.

[50] Wheeler CR, Fried D, Featherstone JD, Watanabe LG, Le CQ ( 2003). Irradiation of dental enamel with Q-switched lambda = 355-nm laser pulses: surface morphology, fluoride adsorption, and adhesion to composite resin. Laser Surg Med.

